# Eavesdropping on dolphins: Investigating the habits of bottlenose dolphins (*Tursiops truncatus*) through fixed acoustic stations

**DOI:** 10.1371/journal.pone.0226023

**Published:** 2019-12-05

**Authors:** Jessica Alessi, Alberta Mandich, Maurizio Wurtz, Chiara Paoli, Carlo Nike Bianchi, Carla Morri, Paolo Povero, Marco Brunoldi, Giorgio Bozzini, Alessandra Casale, Daniele Grosso, Valentina Cappanera, Giorgio Fanciulli, Christian Melchiorre, Gianni Viano, Massimiliano Bei, Nicola Stasi, Mauro Gino Taiuti, Paolo Vassallo

**Affiliations:** 1 Department of the Earth, Environment and Life Science, University of Genova, Genova, Italy; 2 Associazione Me.Ri.S. Mediterraneo Ricerca e Sviluppo, Favara, Agrigento, Italy; 3 CIRCE - Interuniversity Center for Cetacean Research, Operative Unit of Genoa University, Genova, Italy; 4 Department of Physics, University of Genova, Genova, Italy; 5 Area Marina Protetta di Portofino, Ministry for the Environment and for the Protection of Territory and Sea, Santa Margherita Ligure, Genova, Italy; 6 SOFTECO Sismat S.r.l, Genova, Italy; 7 Direzione Marittima di Genova, Ministry for the Infrastructures and Transport, Genova, Italy; Universita degli Studi della Tuscia, ITALY

## Abstract

This study investigates the bottlenose dolphin (*Tursiops truncatus*, Montagu 1821) habitat use in the Portofino marine protected area (NW Italy) and adjacent waters, a core area for the dolphins and a highly touristic area in the Mediterranean Sea. A permanent automated real-time passive acoustic monitoring system, able to detect and track dolphins continuously, was tested in the area within the activities of the Life+ Nature project ARION. The habits of bottlenose dolphins was investigated considering the resident rate inside the area, which quantifies the amount of time dolphins spent in these waters, by means of random forest regression. The dependency of dolphin resident rate was analyzed in relation to four explanatory variables: sea surface temperature, season, time of day, and proximity to the coast. Dolphins spent more time in the area during spring and when sea surface temperature ranged between 15–16°C. Summer resulted the season with lower dolphin residency with significant difference between working day and weekend, in the last the lowest residency was recorded. Main findings provide important information to properly manage the area in order to protect bottlenose dolphins.

## Introduction

Marine mammals are often considered indicators of the health of marine ecosystems and key species in ocean conservation planning [1]; this is because they are used politically to promote reserve designation [2]. Amongst Mediterranean cetaceans, the bottlenose dolphin (*Tursiops truncatus*, Montagu 1821) is highly protected by international policy. The Mediterranean population is listed as a Vulnerable Species under the Red List of Threatened Species category [3] from the International Union for the Conservation of Nature (IUCN) due to its general decline. Since the 1940s a reduction of population was recorded caused by various threats [4] such as: i) loss and degradation; iv) disturbance by marine traffic; v) and high levels of contamination intentional killing, until the 1960s; ii) incidental mortality in fishing gear that continues to be reported; iii) habitat by pollutants. Consequently, the species is listed in the Annex II of the Habitat Directive (Council Directive 92/43/EEC) [5]. This Directive requires Member States to select, designate and protect sites that support specific natural habitats or species as Special Areas of Conservation (SACs). Habitat Directive aim to create a network of SACs across the European Union known as the Natura 2000 Network. The success of SACs in protecting bottlenose dolphin, strictly depends on the available information on the behaviour habits of the species, not only upon distribution for boundaries definition but also to understand how these areas are used by the animals [6]. The knowledge of the factors affecting their presence and residency in an area, is pivotal for protection and management purposes.

The behaviour of the bottlenose dolphin has been studied all around the world with different approaches [6, 7, 8, 9, 10, 11, 12]. However, bottlenose dolphin is a species exhibiting a wide variety of habitat uses and preferences among populations from different geographic areas [13, 14]. The aim of this study is to contribute to bottlenose dolphin conservation investigating their resident rate in the waters off Portofino (NW Italy), a Marine Protected Area (MPA) which represents a core area for bottlenose dolphins [15]. The species is included in the standard form of the SAC named “Fondali Monte di Portofino” that is managed by Portofino MPA but doesn’t have the same boundaries being 198 ha wider than the MPA. Portofino MPA is also subjected to several human activities potentially affecting bottlenose dolphin population such as tourism, fishing activity and intense marine traffic. Portofino MPA extends for 346 ha, and approximately 15000 boats were observed during the summer season, with an average of 250 boats observed during the weekend [16].

Under the framework of the Life+ Nature project called ARION (Life+09 Nat/It/190), in front of Portofino promontory an area was chosen to install and test a permanent automated real-time passive acoustic monitoring system [17]. Inside the *Pelagos Sanctuary* for marine mammals [18], Portofino MPA is recognized as one of the most visited areas by bottlenose dolphins [15, 19], but little information is available regarding how long the dolphins are present and stay in the area. The aim of this study was to investigate factors influencing the time dolphins were present in the area (hereinafter resident rate). Resident rate was defined as minutes recorded with at least one whistle, in order to have an estimation of the time spent by dolphins in the area. In particular, the dependency of the resident rate of the dolphins is analyzed in relation to a set of four explanatory variables: sea surface temperature (SST), season, time slot and proximity to coast (zone hereinafter). Since the Portofino MPA is mostly frequented by tourists during weekend days and the summer season [20], differences in the resident rate of dolphins between working days and weekends for each season were considered, in order to identify effects on dolphins habits potentially useful for management purposes.

## Materials and methods

This study was developed in the framework of the EU funded Life+ Nature project ARION. ARION system is a passive acoustic monitoring tool able to record and to transmit continuously (24 hours/day) bottlenose dolphin whistles.

**Fig 1:**
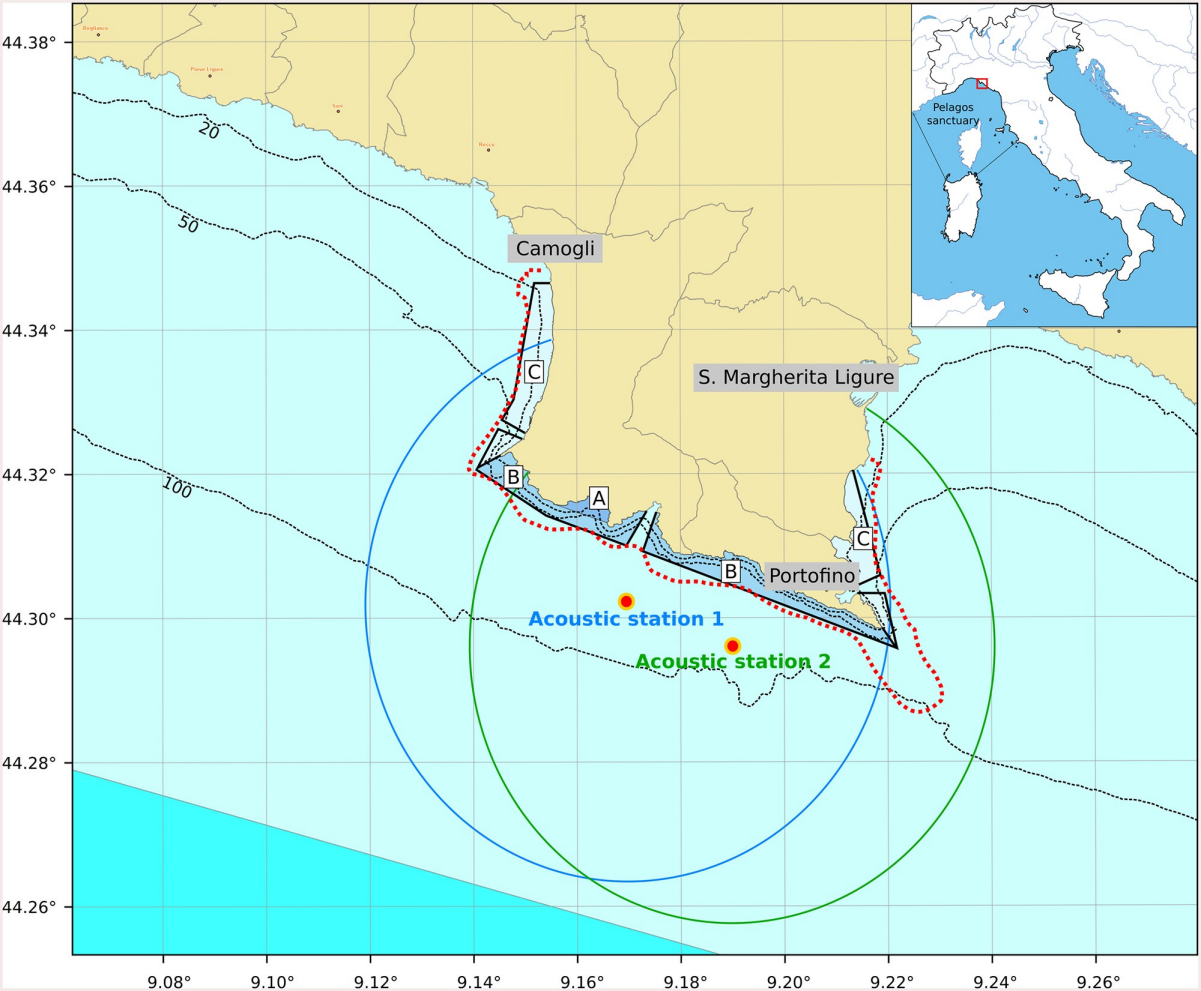
Map of the study area: The boundaries of the Portofino MPA and the position of the three different zones with restricted access are reported on the map (A zone is no entry—No take; B zone is the general reserve; C zone is the partial reserve). The round areas centered on the two acoustic units represent the monitoring area of the system (from [17]). The red dotted line identifies SAC boundaries.

**Fig 2:**
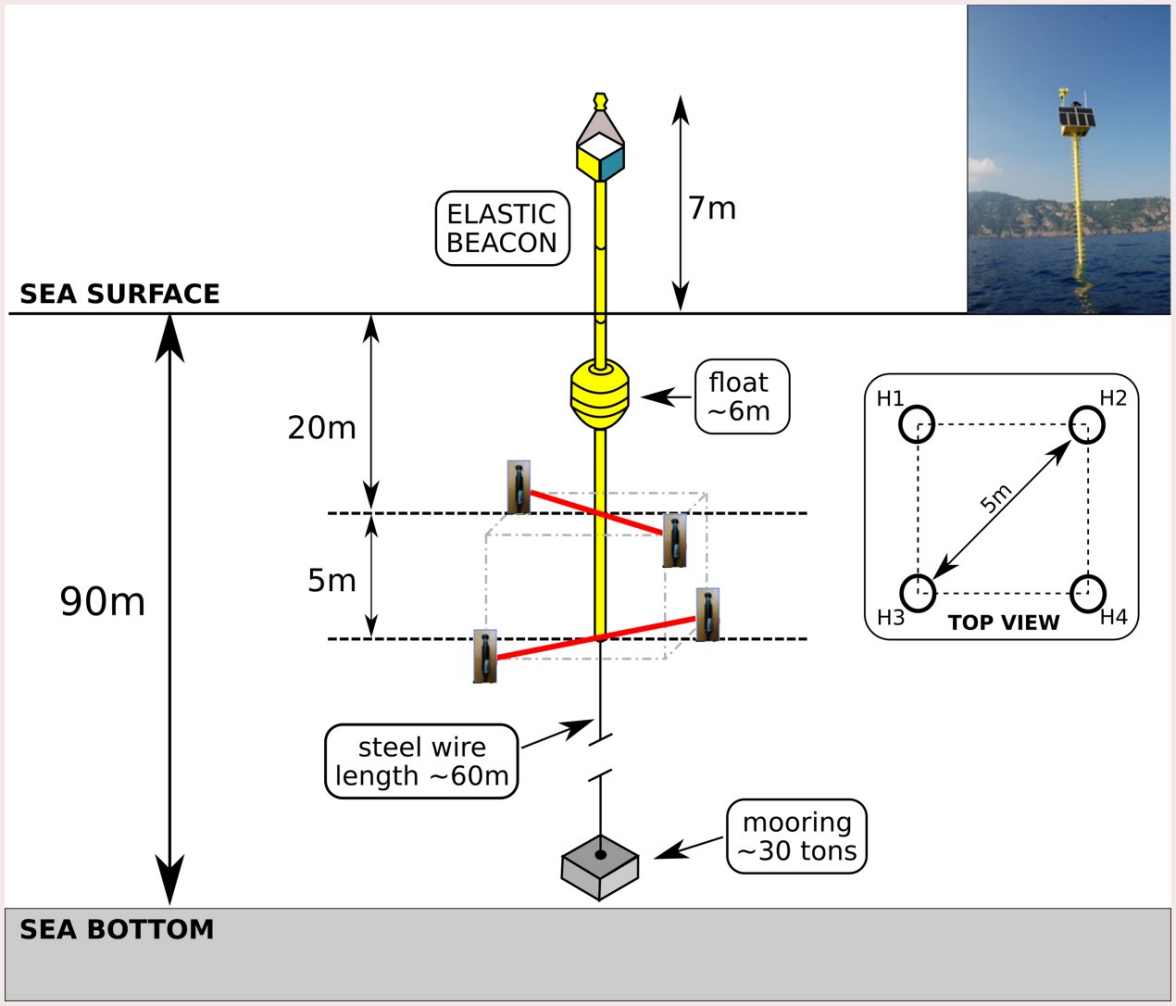
Schematic drawing of the acoustic unit (from [17]).

The system, operating from July 2013 to December 2015, was installed on two elastic beacons in the waters in front of the Portofino MPA, at 1 km from the shoreline (Fig 1), and it was composed by two acoustic recording units each one equipped with four COLMAR GP0280-M omnidirectional hydrophones with a receiving sensitivity of−169 dB re 1 V/μPa; −3dB 740 Hz to 68 kHz (hardware filter: -3dB 3kHz to 23kHz). In particular, the two beacons were located aiming at the dolphins detection within the 'general reserve' zone of Portofino MPA, which includes the largest part of the MPA and occupies the southern front of the promontory [21]. Two pairs of hydrophones were deployed at depths of 20m and 25m respectively on each elastic beacon (Fig 2).

The deployment of the two elastic beacons was authorized by Italian Ministry for Infrastructures and Transports, Liguria Regional Agency (conservation of the regional coastal ecosystem) and Ministry of Cultural Heritage and Activities (underwater prospection for possible presence of archaeological structures or remnants).

The system transferred acoustic data via Wi-Fi link with the land station located in the Portofino lighthouse. Here, data were processed to determine dolphin presence and to track their position in real time thanks to the triangulation method described by Brunoldi and collaborators [17]. The system is able to detect dolphin whistle from 1500 m to 3700 m distance from the beacons, depending on the sea-state [17].

An html file with the summary of the recorded events was automatically generated every six hours. The html files were saved to a local directory but they were visible and accessible also via internet.

The database analyzed in this work reported a record for each time period of six hours. Each record reported 7 entries:

1. the total uptime of the acoustic system, calculated as the minutes of operation of the beacon that has been active for longer time;
2. the number of minutes with at least one whistle of bottlenose dolphin. The whistles were checked one by one by an experienced researcher that observed spectrograms in the html files, in order to avoid false positive records;
3. presence/absence of bottlenose dolphins;
4. resident rate of the dolphins, calculated as the proportion between the number of minutes with at least one whistle and the total uptime;
5. position of the emitted whistles distinguishing between onshore, offshore, or both (when during the six hours period whistles are detected in both areas);
6. season, month and time slot. The seasons were defined as follow: Winter (January–March), Spring (April–June), Summer (July–September) and Autumn (October–December). Four different time slot are considered: i) 00:00–06:00; ii) 06:00–12:00; iii) 12:00–18:00; iv) 18:00–24:00;
7. SST, derived from the probe installed on one of the two elastic beacons. The underwater devices, a Nortek Acoustic Wave and Current (AWAC) profiler and a Idronaut OceanSeven 316+ multiparameter water probe (salinity, conductivity, pH, oxygen level) have been deployed at about 20 and 6 meters depth respectively and fixed to specific underwater supports linked to the main pole

Total and seasonal occurrences of bottlenose dolphins in the area were calculated as the percentage of the number of days with dolphins over the number of days monitored.

Furthermore, resident rate was calculated as the ratio between the number of minutes with at least one whistle detected over the total recording time in minutes for each time slot (6 hours).

### Random forest regression

The dependency of the resident rate of the dolphins was analyzed in relation to a set of four explanatory variables: zone, SST, season, and time slot. As the data set was zero-inflated, the number of absence records was balanced to the number of presence ones. This was done by maintaining all the presences and randomly extracting the corresponding number of absences [19, 22, 23, 24]. Dependency has been investigated by means of Random Forest (RF), a methodology based on regression trees able to model a response variable from a number of explanatory variables by subdividing a dataset into subgroups [25]. This is achieved by two means: (1) a random selection of explanatory variables is chosen to grow each tree and (2) each tree is based on a different random data subset, created by bootstrapping [26]. In this way random forest is able to overcome problems related to the cross-correlation among explanatory variables [27].

Finally, the optimal “splitting” in comparison with real data is identified and selected as a predictor. The rank importance of each explanatory variable is accounted for as the changes in mean square error estimated by leaving a variable out of the model. After the most relevant variables were identified, the following step consisted in exploring the dependence between response variable and each explanatory variable. Thus, partial dependence plots were used to graphically characterize relationships between individual explanatory variables and response obtained from RF [28]. Random forest has been recently applied to cetacean distribution and abundance analyses [19, 23, 29] demonstrating high accuracy and reliability.

### Influence of season and working/weekend days on resident rate

Pleasure boating is one of the main disturbance acting in the area [20]. Data on presence of boats were not available for this study but the effect on dolphin resident rate has been investigated using season and day of the week as proxies of disturbance since pleasure boating in the area is a seasonal activity with presence peaks during weekends [20]. We tested whether the resident rate was significantly different between weekdays and weekends throughout the year using a two-way analysis of variance (ANOVA) taking into consideration season (4 levels: Winter, Autumn, Summer and Spring) and day (2 levels: weekday and weekend). The analysis was performed using code developed and implemented in MATLAB [30].

## Results

**Fig 3:**
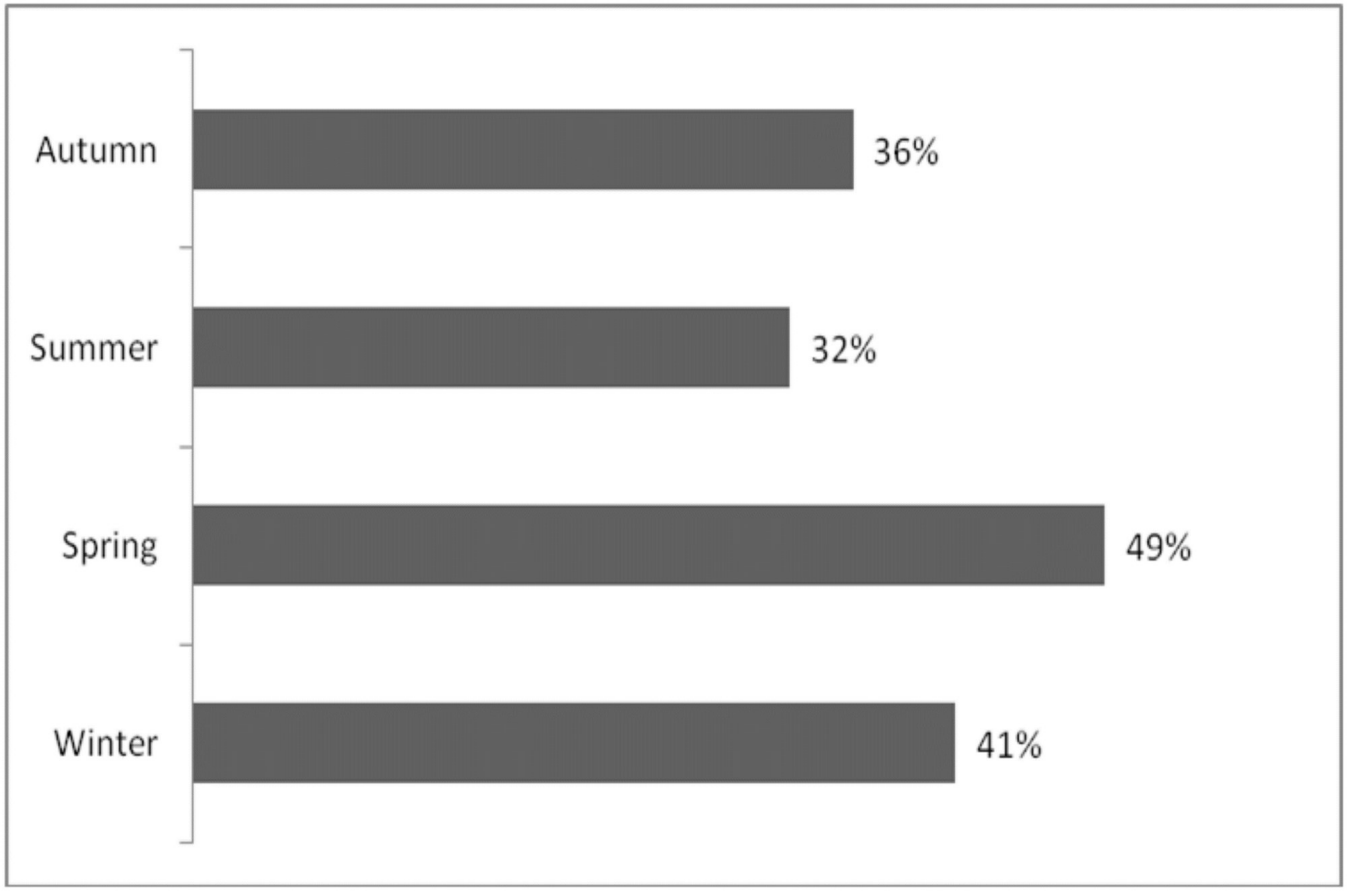
Bottlenose dolphin occurrence per season calculated as percentage of the number of days with dolphins over the number of monitored days.

Arion system was operating from July 8^th^, 2013 to December 24^th^, 2015, being online and recording for a total of 563180 minutes. Over the whole recording period 1711 minutes with at least one whistle were detected. This allows arguing that bottlenose dolphins were actively communicating in the area 0.3% of the time. Bottlenose dolphins were detected during 265 days over 710 (37%), with frequencies slightly variable during seasons (Fig 3) and a more frequent detection during Spring.

The number of detection was not uniformly distributed over years (Table 1): 2014 was the year with the highest number of whistles detected (202), together with the greatest resident rate. The greatest presence of dolphins (286 days with at least one whistle recorded) was recorded during 2015 (Table 1). The resident rate of dolphins in the area varied between years ranging from 0.18% in 2013 to 0.41% in 2014 (Table 1).

**Table 1:** Yearly results of the acoustic monitoring. Yearly occurrence of bottlenose dolphins (Nr. of days with whistles) and respective detection number were reported. Resident rate per year is accounted as the ratio of Minutes with at least one whistle recorded (A) and the total minutes monitored (B).

Year	Nr. of days with whistles	Nr. of detections	Minutes whistle (A)	Minutes monitored (B)	Resident Rate % (A/Bx100)
2013	151	56	53	28652	0.18
2014	273	202	1155	280046	0.41
2015	286	119	503	254482	0.20

**Fig 4:**
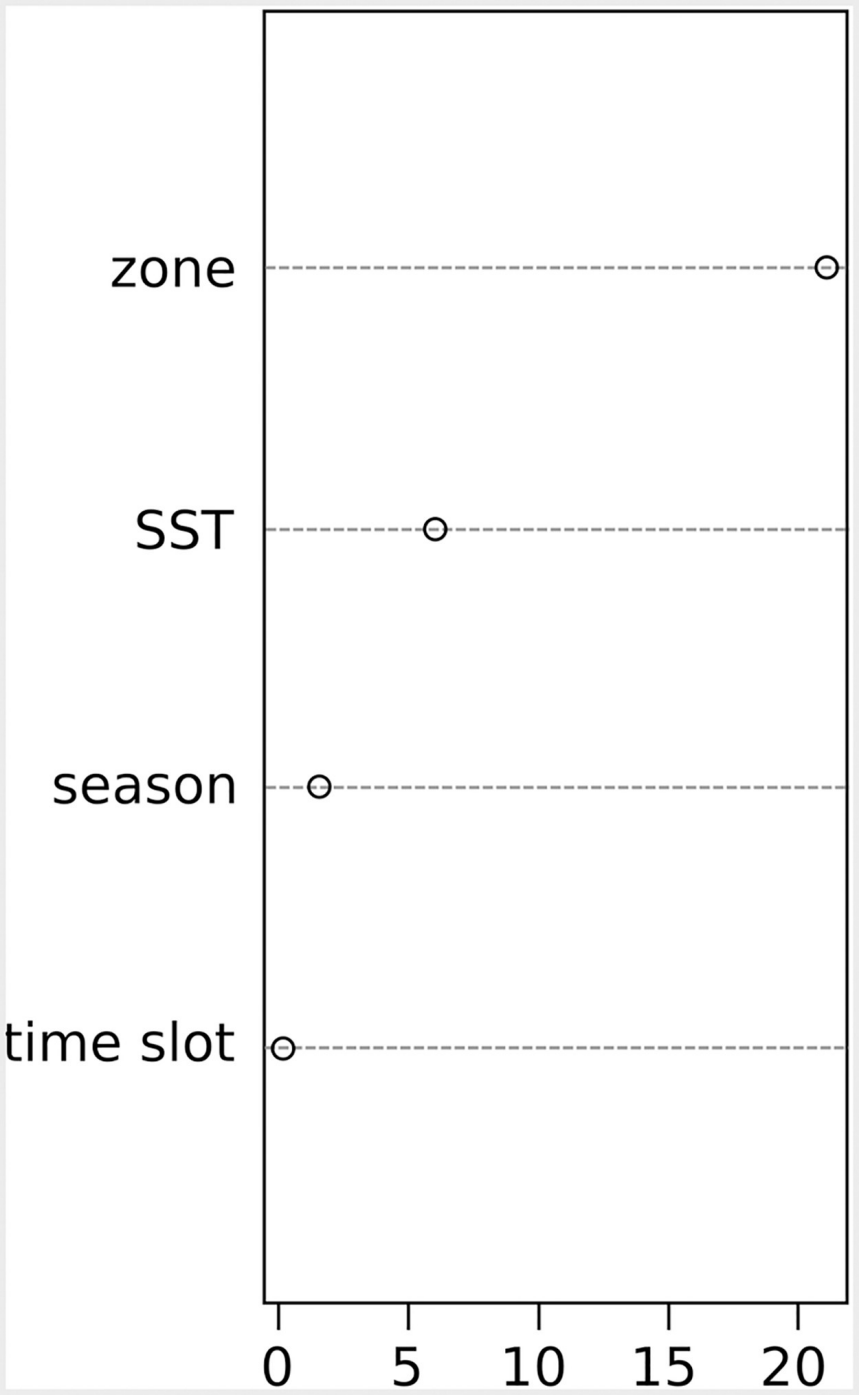
Ranking importance scores of the explanatory variables used in the RF model.

Random forest regression allowed the identification of the influence of each explanatory variable on the resident rate of bottlenose dolphins in the area. The analysis identified the residency of dolphins in Portofino waters mainly dependent on the zone visited, followed by SST, season and, lastly, the time slot (Fig 4).

**Fig 5:**
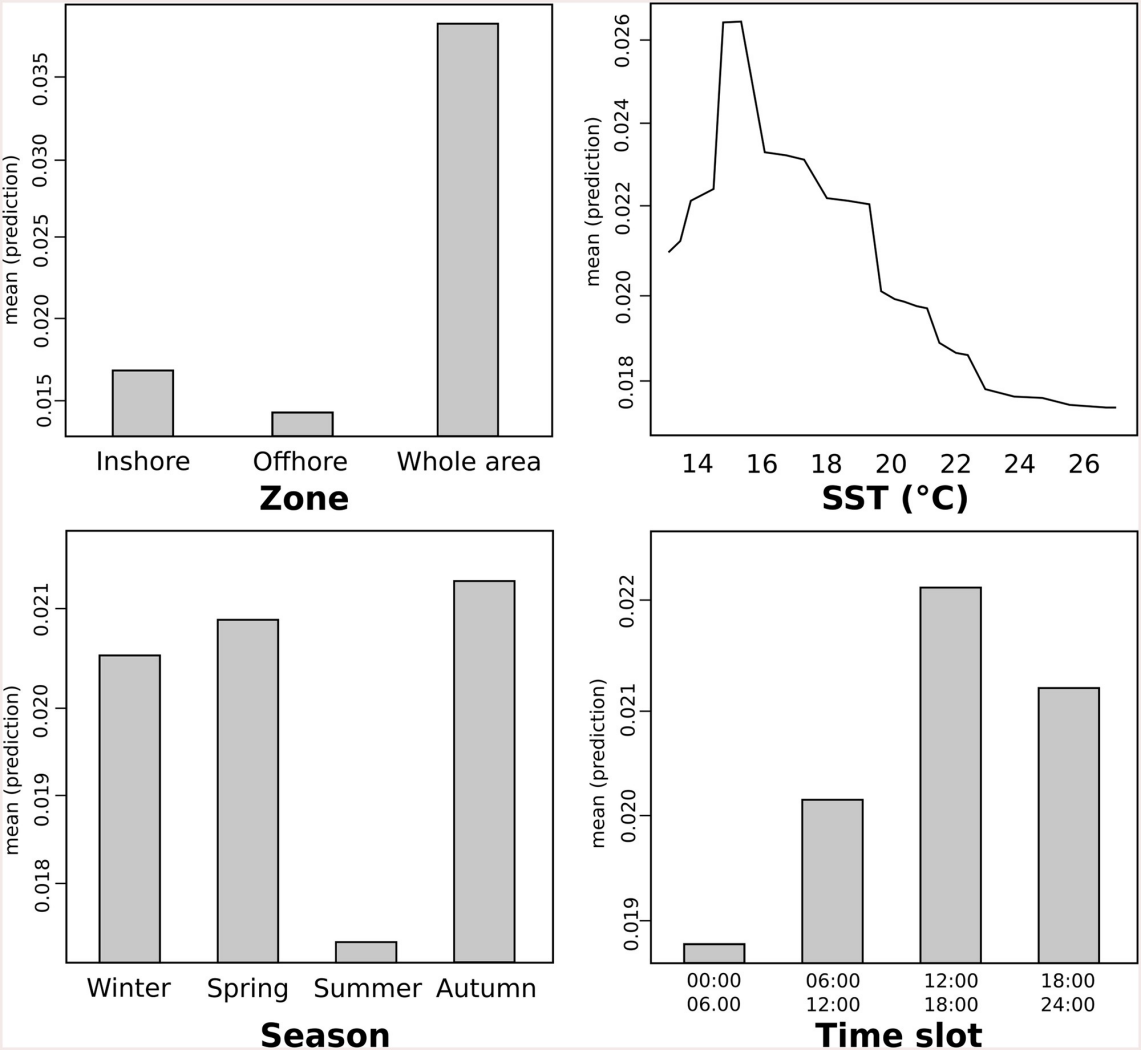
Partial dependence plots of considered explanatory variables.

Bottlenose dolphins spent longer time in the waters in front of Portofino promontory when visiting the whole monitored zone, both inshore and offshore (Fig 5). Resident rate of dolphins resulted strongly influenced also by SST with the highest resident rate at surface temperature around 15–16°C. Higher temperatures display lower resident rates (Fig 5). Summer had the lowest resident rate, while Autumn had the highest. The dolphins stayed longer in the area during the afternoon (12:00–18:00) (Fig 3).

**Fig 6:**
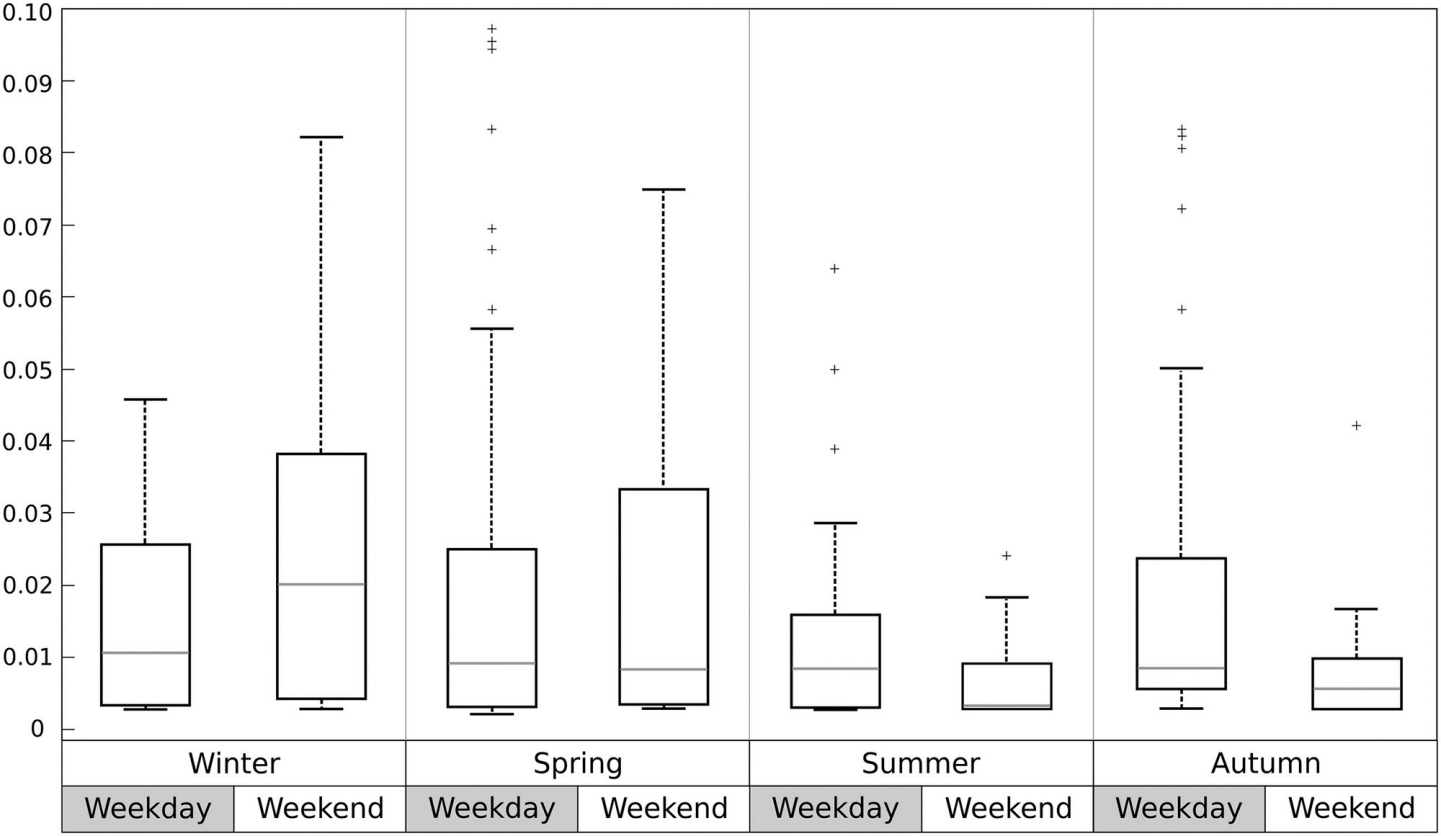
Box plot of resident rate of bottlenose dolphins in the weekday and weekend days during each season.

The bottlenose dolphins stayed longer in the area during Winter (highest median and widest variance) while the minimum resident rate was detected during Summer weekends (lowest median value and narrowest variance) when the pleasure boating activity in the area reaches the maximum level (Fig 6). The differences among resident rates were tested by means of a two-way analysis of variance with interaction between season and day-type (Table 2). In particular, resident rate resulted especially dependent on the cross effect of season and day-type.

**Table 2:** Statistical test (ANOVA) of the effect of season and type of day on the resident rate of bottlenose dolphins in the area.

Source	Sum Sq.	d.f.	Mean Sq.	F	Prob>F
**WE**	0.00025	1	0.00025	0.23	0.6354
**season**	0.00751	3	0.0025	2.22	0.0859
**WE x season**	0.00986	3	0.0033	2.92	**0.034**
**Error**	0.41687	369	0.00113		
**Total**	0.42966	376			

## Discussion

Several studies worldwide have considered species occurrence in order to determine the habitat use of a cetacean in a specified study area (e.g. [11, 31, 32, 33, 34, 35]). Since time and space are fundamental aspects in animal ecology [36], in this work, for the first time, the habits of bottlenose dolphin were studied through the analysis of their residency in the area. The information of how much time dolphins spent in the study area was gained thanks to a permanent and automatic real-time passive acoustic monitoring system in the waters off the Portofino Marine Protected Area (MPA), Ligurian Sea. Use of a fixed acoustic station is highly convenient to investigate the habits of dolphins. It allows to estimate the bottlenose dolphins residency in the area, since it enables collecting continuous data, even in adverse weather conditions and during night, situations in which common sampling methods such as visual surveys do not allow monitoring.

The waters in front of the Portofino MPA resulted highly frequented by dolphins, which were detected in 37% of the recording days. Dolphins were detected during all seasons but more frequently during Spring and less in Summer. Reduced dolphin presence in Summer might be related to many factors, such as food availability or a different vocalization rate, but it could be also due to increased human disturbance, namely, pleasure boat traffic [20]. The time spent in the area was highly variable between years while the annual resident rate remained low during the whole investigation period, suggesting that the area may be used as an ecological corridor to move from western to eastern Ligurian Sea and *vice versa*, in agreement with what previously hypothesized through photo identification [36].

The random forest regression revealed that zone is the most important factor influencing the resident rate. When dolphins stay longer in the area, they occupy all the monitored zone, both inshore and offshore, widely outside MPA boundaries. Higher resident rate are linked to exploration of the whole area, suggesting active movement in the zone probably gathering food rather than travelling along the coast. SST was also an important factor, with the longest residency at 15–16°C and a continuous decreasing trend at increasing temperature. This is likely due to an indirect effect of the SST on prey abundance and not a temperature limit on dolphins themselves, being the species recorded worldwide in all tropical and temperate waters [13]. Similar results were observed in northwest Atlantic, where sighting probability and pod size of transient bottlenose dolphins was dependent on SST, 17°C being the temperature threshold value [37]. The bottlenose dolphin diet in the Ligurian Sea is mainly composed of fish and only secondarily of cephalopods with a ratio of 7:1 [38]. The European hake (*Merluccius merluccius*) is the most hunted species: it was found in 41.5% of the examined stomachs, representing 32.1% of the total number of prey and 30.3% of the total weight of the prey [39]. This species is followed in order of importance in the bottlenose dolphin diet in the Ligurian Sea by *Conger conger* and Sparidae [39]. Research conducted on the feeding behaviour of bottlenose dolphins near Portofino Promontory has revealed strong connection with the following fish species: *Scomber scombrus*, *Boops boops*, *Merluccius merluccius*, *Oblada melanura*, *Sparus aurata*, *Dicentrarchus labrax* [40]. As known, temperature influences fish life at various stages [41]: larval growth and mortality [42, 43], timing of food availability for early ages [44], growth [45], maturity [46], timing of spawning [47] and egg viability [48]; furthermore SST can affect biomass [49]. Probably dolphins stay longer in the area in order to feed, attracted by the presence of prey aggregations. As an example, considering the European hake, which has a great impact in the diet of bottlenose dolphins in the NW Mediterranean Sea, it has been observed that large females and big males tend to aggregate toward the edge of the shelf during Autumn [50], concomitant with the greater residency of dolphins observed in this study.

The resident rate of bottlenose dolphins resulted related to the season and the time slot, spending longer time in the area during Winter and in the afternoon. Although also these dependencies may be linked to food availability, it is also clear that Summer is the season with the highest boat traffic in the studied area due to pleasure crafts, with the highest traffic during the weekend days [20]. The soundscape exhibit strong seasonality in different coastal areas of the Mediterranean Sea [51, 52], furthermore, the presence of numerous boats with erratic conduct could be, plausibly, the cause of low residency of dolphins in that period since both factors are known to be a source of direct disturbance. In fact, has been proved that the number of boat present affect the habits of bottlenose dolphins by reducing the time they spent in resting and milling [53], shifting upward the whistle frequency parameters [54] and changing their surfacing pattern [55]; erratic conduct of the boat affects the inter animal distance and the swimming speed of dolphins [56]. When intense boat traffic occurs, bottlenose dolphins could avoid temporary favorable areas, confirming what observed in many cases worldwide [57, 58, 59, 60, 61]. This hypothesis has been further tested considering the resident rate differences between weekdays and holidays per season. During the weekends, and only during Summer, boat traffic increases considerably, until four times more than week days, and in concomitance the dolphins resident rate decreases. These changes in residency are statistically different, as proved by two-way analysis of variance. Nevertheless, further analysis considering boat traffic, both in terms of numerousness and noise level produced, are necessary to confirm the effect of anthropogenic disturbance on the residency of bottlenose dolphins in the waters of the Marine Protected Area of Portofino. Additionally to noise disturbance, marine traffic has negative effects on the survival of cetaceans, various species have been recorded dead or alive with evidence of propeller wounds in the Pelagos Sanctuary [62, 63]. In conclusion, this work confirms the importance of the study area for bottlenose dolphin conservation in the Mediterranean, as underlined in previous studies [15]. From a management perspective, an enlargement of Portofino MPA towards offshore waters is highly recommended because of the presence of dolphins in both inshore and offshore waters. An extension of MPA boundaries could guarantee the improvement of management measures and the boat traffic regulation. This has been tested during ARION lifespan with the release of a protocol of conduct for reducing risks for the species. Specifically, the ship and boat owners present in the area had to follow a protocol of conduct with several regulations such as to reduce vessels speed (lover than 5 knots), avoid heading change, and keep a safe distance from the animals. This approach should ensure the species protection improvement, the sustainable coexistence of dolphins and anthropic activities and will promote responsible usage of the sea.

## Supporting information

S1 Dataset In the dataset were reported: The date of monitoring; the hour of starting monitoring; the total uptime of the acoustic system (minutes); the sea surface temperature (SST); the position of the emitted whistles distinguishing between onshore (code 1), offshore (code 2), both (code 3) or not reconstructed position (code 0); the number of minutes with at least one whistle of bottlenose dolphin; and the presence (1)/absence(0) of bottlenose dolphins. (XLSX)
